# The effect of the addition of microbial transglutaminase before the fermentation process on the quality characteristics of three types of yogurt

**DOI:** 10.1007/s10068-019-00640-6

**Published:** 2019-07-04

**Authors:** Małgorzata Ziarno, Dorota Zaręba

**Affiliations:** 1 Division of Milk Biotechnology, Department of Biotechnology, Microbiology and Food Evaluation, Faculty of Food Sciences, Warsaw University of Life Sciences - Szkoła Główna Gospodarstwa Wiejskiego (WULS-SGGW), Nowoursynowska Str. 159C, 02-776 Warsaw, Poland; 2 Secondary Food Industry and Gastronomy Technical School in Warsaw, Komorska Str. 17/23, 04-161 Warsaw, Poland

**Keywords:** Transglutaminase, Fermentation, Syneresis, Lactic acid bacteria, Texture

## Abstract

The effect of the addition of microbial TGase to milk on selected physical properties of the final product and the viability of lactic acid bacteria cultures during storage at 6 °C for 56 days was studied. Three types of set-style yogurt were made with varying parameters. Weekly analyses included the determination of syneresis and water-holding capacity, texture, pH, and the lactic acid bacteria population. Our research has confirmed that mTGase may be used to stabilize yogurts, although the syneresis, the water-holding capacity of yogurts, and the textural features of yogurts were dependent on the step in the production process at which mTGase was added to milk. The presence of mTGase had no relevance with regard to the acidity of yogurts stored under refrigerated conditions. The addition of mTGase had no effect on lactobacilli, but had a variable effect on *Streptococcus thermophilus*, depending on the duration of enzymatic activity.

## Introduction

Lactic acid fermentation is a process that forms the basis for the production of fermented drinks, including yogurts. The physical and structural properties of the resulting gels are determined by both, process parameters (temperature; time; mechanical factors such as stirring, pumping, and aeration) and changes in milk proteins in the stages preceding and following the fermentation (Lucey, 2004). Moreover, each of these factors can contribute to a reduction of the stability of the curd in the finished products. This leads to separation of the curds and whey, which is an unacceptable effect for consumers. Methods to avoid whey syneresis are the enrichment of milk with dry matter, increase of the protein content, or addition of hydrocolloids. The use of transglutaminase (TGase) is one of the more innovative methods for the stabilization of yogurt gel. This enzyme catalyzes the ligation of the amino residues of the glutamine with the ε-amino group of lysine (Abou-Soliman et al., 2017; Ozer et al., 2007; Schorsch et al., 2000a). At present, microbial transglutaminase (mTGase) isolated from *Streptoverticillium mobaraense* is the most frequently used enzyme in the food industry (Farnsworth et al., 2006; Schorsch et al., 2000a; 2000b). According to current literature, the crosslinking of milk proteins by mTGase increases the viscosity of yogurt gel and reduces the occurrence of syneresis (Farnsworth et al., 2003; Lauber et al., 2000a; 2000b; Lorenzen et al., 2002). The effect of TGase on the physical properties of yogurt has been studied by many researchers (Guyot and Kulozik, 2011; Jaros et al., 2010; Lorenzen, 2000; Ozer et al., 2007; Oner et al., 2008). Moreover, the use of TGase creates the possibility of reducing the quantity of milk protein that is added to stabilize the yogurt curd. TGase can be used to modulating yogurt quality (Gharibzahedi and Chronakis, 2018). TGase affects the structure and consistency of milk proteins during the production of yogurts, similar to the effect of structure-building additives (e.g., hydrocolloids) or milk proteins. The crosslinking of milk proteins through TGase similarly (although the mechanism of action is different) improves the physical properties of the yogurts—it prevents syneresis or makes the texture firmer and softer. Bonisch et al. (2007) showed that TGase loses its activity due to a reduction of pH and is not active in the final product (TGase exhibited optimum activity at pH 6.0). This means that the enzyme can be added to milk with starter cultures and there is no need to modify the production process (Bonisch et al., 2007). The time required to achieve the low-pH–inactivating enzyme was sufficient to enable the operation of the enzyme on milk proteins. On the other hand, some investigators recommend heating milk prior to the addition of TGase, because milk may have TGase inhibitors, and heat treatment may improve the reactivity of whey proteins similar to that of milk after termination of mTGase activity for enzyme inhibition (Abou-Soliman et al., 2017; De Jong et al., 2003; Neve et al., 2001). This does not mean, however, that the enzyme does not subsequently affect the final product. An important quality characteristic of yogurt is the adequate level of lactic acid bacteria, especially probiotic strains. Neve et al. (2001) observed that mTGase decreases the viability rate of *Streptococcus thermophilus* and *Lactobacillus delbrueckii* subsp. *bulgaricus* in the first two weeks of yogurt storage. This inhibition of bacterial growth may be due to the low-molecular-weight peptides or amino acids required for bacterial growth that are cross-linked by the enzyme and partially inaccessible to bacterial cells (Faergemand et al., 1999).

This study was conducted to determine the effect of the addition of microbial TGase to milk, either before fermentation (with or without heat treatment at 80 °C prior to fermentation) or concurrently with the starter culture (immediately prior to fermentation) on selected physical properties of the final product and the viability of starter cultures (*S. thermophilus* and *L. delbrueckii* subsp. *bulgaricus*) during storage at 6 °C for 56 days.

## Materials and methods

### Materials

Cow’s milk with a fat content of 3.2% was the raw material to obtain the experimental set-style yogurts. Yogurts were prepared using Yo-S freeze-dried yogurt starter culture (MeB, Italy) containing *Str. thermophilus* and *Lb. delbrueckii* subsp. *bulgaricus*. The starter culture was used in the amount of 0.02% (according to the producer’s protocol). The non-fat solid mass (NFSM) of milk was increased by the addition of skimmed milk powder (SM Gostyń, Poland) in the amount of 1%. Saprona 1L mTGase preparation (All Taste, Poland) was used in the production of yogurts in the amount of 0.04% (according to the producer’s protocol, i.e. 1.5 U/g protein for the standardization of protein content in milk to the level of 6.5%).

### Yogurt samples preparation

Samples of set-style yogurts were produced on a laboratory scale, and four types of yogurts were produced: S1, S2, S3, and C. The S1 yogurts were obtained from milk with SM powder and mTGase added at 12 h prior to fermentation and stored at 6 °C until fermentation. Then, milk with additives was heated to 45 °C immediately prior to fermentation and a starter culture was added (such sample preparation, however not practical, was to check whether the enzyme addition in advance would change the yogurt parameters tested). The S2 yogurts were obtained from milk with SM powder added at 12 h prior to fermentation and stored at 6 °C until fermentation. Then, milk was heated to 45 °C immediately prior to fermentation and mTGase and a starter culture were added. The S3 yogurts were obtained from milk with SM powder and mTGase added at 12 h prior to fermentation and stored at 6 °C until fermentation. Next, milk with additives was heated to 80 °C just immediately prior to fermentation, then cooled down to 45 °C and a starter culture was added. The control samples (C) were the last type of yogurts produced. These were obtained from milk with SM powder added at 12 h prior to fermentation and stored at 6 °C until fermentation. Then, milk with additives was heated to 45 °C just before the fermentation and a starter culture was added.

In each case, the fermentation was conducted at 43 °C for 4–5 h in sterile jars with a capacity of 170 mL. After the end of fermentation, all yogurt samples were cooled down to 6 °C and kept under these conditions for 56 days. Such a long duration of experiments was consciously selected to check the characteristics of the yogurts’ properties; not only those traditionally considered in scientific research (generally yogurt has a short shelf life of about 21 or 30 days), but also in a period far beyond this time range to observe the potential for extending the shelf life of yogurt samples free from yeast recontamination (Lourens-Hattingh and Viljoen, 2002). This is also in line with the expectations of large food retail chains looking for products with ever-longer shelf-life.

### Yogurt samples analyses

The samples for analysis were collected at weekly intervals. The analysis of yogurt samples included: syneresis and WHC (water-holding capacity), texture analysis (hardness, adhesive force and adhesiveness), and pH value determination. Each determination was performed in two parallel repetitions for each analysis, with three independent replications of each experiment.

### Syneresis and water holding capacity (WHC)

The syneresis of yogurt samples was determined according to Farnsworth et al. (2006). The samples were centrifuged at 640 × g for 10 min at 4 °C, than the supernatant was collected and weighed. The water-holding capacity was calculated as: WHC (%) = (m_1_/m_2_) × 100, where m_1_ is the mass of precipitate after centrifugation (g), and m_2_ is the mass of the yogurt sample (g).

### Hardness, adhesive force and adhesiveness

A Brookfield CT3 10 K Texture Analyzer (Brookfield Engineering Laboratories Inc., USA) was used to measure the hardness, adhesive force and adhesiveness of yogurts. A TA4/1000 probe (cylinder, 38.1 mm D, and 20 mm L) was used to measure the texture properties of samples in 170 mL cups at 4 °C. Texture Analyzer Test Settings were as follows: mode normal, trigger 4.5 g, distance 30 mm, speed 1 mm/s.

### Acidity (pH)

The pH value of milk samples was measured with a CPO-505 digital pH-meter (Elmetron, Poland) calibrated at 25 °C with temperature compensation.

### Lactic acid bacteria population

Further analyses included the determination of the lactic acid bacteria population in yogurt using the standard plate method, in two parallel replicates for each analysis and three independent replications for each experiment. MRS agar (Merck, Germany) was used to determine the lactobacilli counts, and M17 agar (Merck, Germany) was applied to determine the streptococci counts. The plates of MRS agar were incubated at 37 °C for 72 h under anaerobic conditions (Anaerocult A, Merck, Germany). Plates of M17 agar were incubated at 37 °C for 72 h under aerobic conditions. After incubation, the number of bacteria was converted into colony-forming units in mL (CFU/mL) and expressed as log CFU/mL.

### Statistical analysis

The mean values were compared using Tukey HSD’s ANOVA at a statistical significance set at *p* < 0.05 (Statgraphics Centurion XV, Statpoint Technologies Inc., USA). To assess the possibility of sample classification based on the survival of bacteria in samples during storage, Principal Component Analysis (PCA) was performed. The statistical analyses were conducted with Statistica v.12 software (StatSoft Inc., USA).

## Results and discussion

All tested textural attributes were found to be dependent on the presence of mTGase (*p *<* 0.05* for each factor), as well as the storage time of yogurts (*p *<* 0.05*). The initial hardness values differed in a significant manner in the samples tested (*p *<* 0.05*). Control samples were characterized by mean hardness values 207 g, whereas higher values were obtained for samples with mTGase (Table 1). These values were significantly higher (*p *<* 0.05*) in the case of S1 and S3 samples than in the control yogurts. No correlation was seen between the populations of lactic acid bacteria or pH values and the textural attributes that were measured. With time, no significant changes in the hardness of control yogurts were observed (*p *>* 0.05*); however, for samples with mTGase, a significant increase in the hardness value cycle was observed as early as 7–14 days of the experiments (*p *<* 0.05*). The smallest changes were observed in S3 and S2 yogurt samples, whereas the greatest changes were reported in S1 samples. The observed and changes can be explained by differences in the duration of mTGase activity in the milk used to prepare different types of yogurts.

**Table 1:** Hardness, adhesive force, and adhesiveness of yogurt samples

Storage time (day)	Hardness (g)							
	C samples		S1 samples		S2 samples		S3 samples	
	Mean	± SD	Mean	± SD	Mean	± SD	Mean	± SD
0	207	± 43.7^a A^	350	± 4.6^b A^	253	± 2.9^a,c A^	261	± 5.2^c A^
7	233	± 22.7^a A^	388	± 2.3^b A,B^	318	± 2.9^c B^	255	± 0.6^a A^
14	202	± 47.6^a A^	433	± 62.9^b B,C,D^	331	± 1.0^c B,C^	318	± 6.9^c B^
21	215	± 43.9^aA^	421	± 2.3^b B,C^	320	± 0.6^c B^	321	± 6.4^c B^
28	241	± 39.2^a A^	472	± 5.8^b C,D,E^	314	± 5.8^c B^	345	± 21.4^c B^
35	227	± 50.0^a A^	483	± 6.9^b D,E^	328	± 8.1^c B,C^	380	± 11.5^c B^
42	225	± 55.7^a A^	513	± 14.4^b E,F^	361	± 5.8^c D,E^	389	± 1.7^c C^
49	218	± 51.9^a A^	548	± 5.8^b F^	374	± 5.8^c E^	342	± 24.0^c B^
56	217	± 4.4^a A^	548	± 5.8^b F^	343	± 21.4^c C,D^	342	± 24.0^c C^

Storage time (day)	Adhesive force (g)							
	C samples		S1 samples		S2 samples		S3 samples	
	Mean	± SD	Mean	± SD	Mean	± SD	Mean	± SD
0	67.2	± 10.46^a A^	100.5	± 0.58^b A,B^	79.5	± 1.73^c A,B,D^	71.0	± 1.15^a,c A^
7	72.0	± 15.30^a A^	119.0	± 1.15^b B^	84.5	± 1.35^a B,C^	74.5	± 5.20^a A,B^
14	67.5	± 21.08^a A^	117.0	± 1.15^b B^	87.0	± 2.31^a C^	81.0	± 1.15^a B,C^
21	69.3	± 17.07^a A^	106.0	± 5.77^b A^	87.5	± 2.89^a,b C^	84.5	± 2.89^a,b C^
28	69.7	± 18.41^a A^	99.8	± 10.10^b A,C^	76.5	± 2.89^a,b D^	81.5	± 2.89^a,b B,C^
35	67.8	± 19.47^a A^	100.5	± 1.73^b A,C^	74.5	± 0.58^a D^	83.0	± 3.46^a,b C^
42	67.5	± 17.63^a A^	100.0	± 1.15^b A,C^	80.0	± 1.15^a,b A,B^	84.5	± 0.58^a,b C^
49	61.7	± 9.14^a A^	95.0	± 2.31^b C^	80.5	± 0.58^c A,B^	79.8	± 4.92^cB,C^
56	56.5	± 0.55^a A^	95.0	± 2.31^b C^	79.3	± 3.77^c A,B,D^	79.8	± 4.91^c B,C^

Storage time (day)	Adhesiveness (mJ)							
	C samples		S1 samples		S2 samples		S3 samples	
	Mean	± SD	Mean	± SD	Mean	± SD	Mean	± SD
0	15.6	± 2.37^a A^	23.4	± 0.46^b A^	15.8	± 0.17^a A,B,C^	15.8	± 0.87^a A,B,C^
7	16.0	± 1.70^a A^	20.7	± 0.40^b B,C^	15.8	± 0.92^a A,B,C^	14.5	± 0.52^a A^
14	15.1	± 3.00^a A^	21.6	± 0.46^bC^	16.8	± 0.47^a C^	16.2	± 1.39^a B,C^
21	14.9	± 1.04^a A^	18.8	± 0.23^b D,E^	17.0	± 0.06^c C^	15.1	± 0.06^a A,B^
28	15.1	± 2.42^a A^	18.6	± 0.12^b D,E^	14.7	± 0.17^a A^	16.7	± 0.75^a,b C^
35	14.3	± 1.38^a A^	18.0	± 1.10^b E^	15.3	± 0.35^a,c A,B^	17.3	± 0.29^b,c C^
42	13.8	± 2.30^a A^	18.0	± 0.12^b E^	15.8	± 0.35^a,b A,B,C^	16.9	± 0.23^a,b C^
49	13.1	± 1.20^a A^	19.4	± 0.52^b B,D^	16.4	± 0.46^c B,C^	17.1	± 0.17^c C^
56	13.3	± 0.82^a A^	18.9	± 0.82^b D,E^	15.8	± 0.89^c A,B,C^	17.1	± 0.17^c C^

C samples—obtained from milk with skimmed milk powder added at 12 h prior to fermentation and stored at 6 °C until fermentation, then a starter culture was added to milk prior to fermentation

S1 samples—obtained from milk with skimmed milk powder and the mTGase added at 12 h prior to fermentation and stored at 6 °C until fermentation, then a starter culture was added to milk prior to fermentation

S2 samples—obtained from milk with skimmed milk powder added at 12 h prior to fermentation and stored at 6 °C until fermentation, then mTGase and a starter culture were added immediately prior to fermentation

S3 samples—obtained from milk with skimmed milk powder and mTGase added at 12 h prior to fermentation and stored at 6 °C until fermentation, then milk was heated to 80 °C and a starter culture was added after cooling down to 45 °C before the fermentation process

^a, b, c^Significant differences in each row among the means (for *p* < 0.05)

^A, B, C, D^Significant differences among the means (in each column for each parameter) (for *p* < 0.05)

Mean ± SD = mean values ± standard deviations of six experiments

Similar correlations and trends were found for the adhesive force (Table 1). Values of the adhesive force in fresh yogurts differed significantly based on the presence of mTGase (*p *<* 0.05*). The greatest adhesive force values were recorded in S1 samples, and these significantly differed from the values determined for control, S2, and S3 yogurts. The measurements undertaken from week to week demonstrated that different activities of mTGase resulted in different changes in the values of adhesive force in the various samples of stored yogurts. These changes differentiated yogurt samples in a similar way, as was observed in the case of syneresis, WHC, and hardness cycle. Starting from day 14 of experiments, the adhesive force value of the stored samples was significantly lower than in fresh S1, S2, and S3 yogurts (*p *<* 0.05*). These values further decreased with storage time. No significant changes were found in the case of control samples *p *>* 0.05*. The measurement of adhesive force confirmed the impact of mTGase activity in milk before or during fermentation.

Furthermore, adhesiveness showed a significant influence of mTGase (*p *<* 0.05*) and storage time (*p *<* 0.05*). The adhesiveness of the fresh S1 yogurts was significantly higher than that in the other samples (*p *<* 0.05;* Table 1). This means that longer period of mTGase activity (as in S1 samples compared to other yogurt samples) substantially affected the adhesiveness value, regardless of pH values. Moreover, the storage time in refrigerated conditions had a significant importance for the adhesiveness value, but only in the case of yogurt produced from milk with added enzymes. There was no correlation between adhesiveness and the populations of lactobacilli or streptococci. The adhesiveness of yogurts decreased with time, and the changes were significant from days 7 to 14 of the experiments (*p *<* 0.05*). The S2 yogurts were an interesting case. Significant changes in the adhesiveness of these samples were reported until the day 28 of storage; on subsequent days, the adhesiveness did not differ significantly from the values measured in fresh yogurts. These values were significantly different when compared to those for the control samples; however, they did not significantly differ as compared to values obtained for fresh yogurts. According to these results, allowing sufficient time for the enzymatic reaction with milk proteins resulted in significant changes in the textural features of yogurts over a 56-day storage period. Therefore, it appears that the most important and essential condition for the full reaction of mTGase with milk proteins is storage time before fermentation.

Immediately after the end of fermentation, the pH depended significantly on the type of sample (*p *<* 0.05*). The pH of S2 samples significantly differed from that of the control, S1, and S3 samples (Table 2). The differences observed in the initial acidity of yogurts were reflected in the difference in the level of streptococcal populations. Thus, the effect of mTGase on the acidity of yogurts immediately after their production and its correlation with the population of *S. thermophilus* were demonstrated. The pH value of yogurts gradually decreased during further refrigerated storage, and these changes were significant (*p *<* 0.05*) from days 21 to 28. Moreover, the population of *S. thermophilus* differed between samples in the same period, and the differences between the acidity of stored yogurts diminished with storage time. On the final day of the experiment, pH values did not depend on the type of sample. This means mTGase had no relevance with regard to the acidity of yogurts stored under refrigerated conditions.

**Table 2:** The pH, syneresis, and WHC values of yogurt samples

Storage time (day)	pH value							
	C samples		S1 samples		S2 samples		S3 samples	
	Mean	± SD	Mean	± SD	Mean	± SD	Mean	± SD
0	4.64	± 0.013^a A^	4.61	± 0.010^b A^	4.67	± 0.002^c A^	4.60	± 0.021^b A^
7	4.58	± 0.042^a A,B^	4.55	± 0.001^a A,B^	4.60	± 0.056^a A,B^	4.60	± 0.051^a A^
14	4.57	± 0.044^a A,B^	4.47	± 0.007^b B,C^	4.49	± 0.058^a,b A,B,C^	4.47	± 0.046^b B^
21	4.55	± 0.041^a A,B^	4.45	± 0.001^b B,C^	4.48	± 0.062^a,b A,B,C^	4.41	± 0.002^b B,C^
28	4.50	± 0.056^a B,C^	4.39	± 0.051^a C^	4.51	± 0.111^a A,B,C^	4.40	± 0.111^a B,C^
35	4.44	± 0.070^a C^	4.38	± 0.053^a C,D^	4.45	± 0.059^a B,C^	4.43	± 0.057^aB,C^
42	4.41	± 0.055^a C^	4.27	± 0.064^b E,F^	4.45	± 0.067^a B,C^	4.38	± 0.000^a B,C^
49	4.31	± 0.074^a D^	4.29	± 0.068^a E,D^	4.38	± 0.084^a C,D^	4.34	± 0.013^a C^
56	4.20	± 0.059^a E^	4.16	± 0.059^a F^	4.25	± 0.161^a D^	4.16	± 0.048^a D^

Storage time (day)	Syneresis (%)							
	C samples		S1 samples		S2 samples		S3 samples	
	Mean	± SD	Mean	± SD	Mean	± SD	Mean	± SD
0	12.7	± 1.92^a A^	10.8	± 0.80^a,b A^	10.4	± 0.31^a,b A^	8.5	± 1.14^b A^
7	16.0	± 0.94^a B^	25.8	± 1.31^b B^	11.6	± 0.51^cA^	12.1	± 2.22^d B^
14	17.9	± 0.78^a B,C^	28.3	± 0.63^b C^	15.3	± 1.13^c B^	16.8	± 1.27^a,c B,C^
21	20.0	± 0.54^a C^	29.6	± 0.86^b C,D^	18.4	± 1.74^a C^	18.1	± 1.92^a C^
28	22.7	± 1.19^a D^	30.9	± 0.74^b C,D,E^	22.3	± 1.09^a D^	21.6	± 0.57^c D^
35	28.5	± 2.44^a E^	31.4	± 0.80^b D,E,F^	25.6	± 0.77^a D,E^	22.1	± 0.32^c D,E^
42	33.6	± 1.19^a F^	31.6	± 0.80^a E,F^	25.7	± 0.41^b D,E^	23.3	± 1.10^c D,E,F^
49	35.5	± 0.32^a G^	33.7	± 0.99^b F,G^	26.1	± 0.56^c E,F^	24.0	± 0.91^d E,F^
56	36.9	± 0.92^a G^	33.7	± 0.80^bG^	27.8	± 0.71^c F^	25.4	± 1.22^cF^

Storage time (day)	WHC (%)							
	C samples		S1 samples		S2 samples		S3 samples	
	Mean	± SD	Mean	± SD	Mean	± SD	Mean	± SD
0	87.3	± 1.92^a A^	89.2	± 0.80^a,b A^	89.6	± 0.31^a,b A^	91.5	± 1.14^b A^
7	84.0	± 0.94^a B^	74.2	± 1.31^b B^	88.4	± 0.51^c A^	87.9	± 2.22^d B^
14	82.1	± 0.78^a B,C^	71.7	± 0.63^b C^	84.7	± 1.13^c B^	83.2	± 1.27^a,c B,C^
21	80.0	± 0.54^a C^	70.4	± 0.86^b C,D^	81.6	± 1.74^a C^	81.9	± 1.92^a C^
28	77.3	± 1.19^a D^	69.1	± 0.74^b C,D,E^	77.7	± 1.09^a D^	78.4	± 0.57^c D^
35	71.5	± 2.44^a E^	68.6	± 0.80^b D,E,F^	74.4	± 0.77^a D,E^	77.9	± 0.32^c D,E^
42	66.4	± 1.19^a F^	68.4	± 0.80^a E,F^	74.3	± 0.41^b D,E^	76.7	± 1.10^c D,E,F^
49	64.5	± 0.32^a G^	66.3	± 0.99^b F,G^	73.9	± 0.56^c E,F^	76.0	± 0.91^d E,F^
56	63.1	± 0.92^aG^	66.3	± 0.80^b G^	72.2	± 0.71^c F^	74.6	± 1.22^c F^

C samples—obtained from milk with skimmed milk powder added at 12 h prior to fermentation and stored at 6 °C until fermentation, then a starter culture was added to milk prior to fermentation

S1 samples—obtained from milk with skimmed milk powder and the mTGase added at 12 h prior to fermentation and stored at 6 °C until fermentation, then a starter culture was added to milk prior to fermentation

S2 samples—obtained from milk with skimmed milk powder added at 12 h prior to fermentation and stored at 6 °C until fermentation, then mTGase and a starter culture were added immediately prior to fermentation

S3 samples—obtained from milk with skimmed milk powder and mTGase added at 12 h prior to fermentation and stored at 6 °C until fermentation, then milk was heated to 80 °C and a starter culture was added after cooling down to 45 °C before the fermentation process

^a, b, c^Significant differences in each row among the means (for *p* < 0.05)

^A, B, C, D^Significant differences among the means (in each column for each parameter) (for *p* < 0.05)

Mean ± SD = mean values ± standard deviations of six experiments

It is known that the acidity of fermented milks is the key factor affecting the amount of whey that is separated (Lee and Lucey 2010). The lower pH value, the higher the degree of syneresis. In addition, the process of whey syneresis is associated with such factors as protein denaturation as well as the method and intensity of milk heat treatment. The syneresis and WHC of our samples depended on the presence of mTGase (*p *<* 0.05*) and the storage time of the samples (*p *<* 0.05*). All yogurt samples produced from MTGase-treated milk had a greater capacity for WHC, and whey separation significantly decreased. Immediately after the end of fermentation, syneresis or WHC of S3 yogurts significantly differed from that of the control yogurts; however, S1 and S2 samples did not show such differences (Table 2). The S3 samples were characterized by a lower syneresis value and a greater WHC value than the control yogurts. This means that the timing of mTGase addition and the method of milk preparation after enzymatic activity are significant for the tested parameters of the obtained yogurts. These differences persisted even during refrigerated storage of samples. There was no correlation between the changes in the population of lactic acid bacteria or pH value and changes in syneresis or WHC of yogurts. A significant increase in syneresis and a decrease in the WHC value were observed after 7–14 days of storage. Syneresis in control yogurts averaged 36.9% on the last day of analysis; however, in S1 samples, it was lower than in the control yogurts and averaged 33.7%, whereas it was only 25.4–27.8% in S2 and S3 samples. In terms of the WHC, the control, S1, S2, and S3 yogurts were characterized by average values of 63.1%, 66.3%, 72.2%, and 74.6%, respectively. The values of syneresis and WHC were stable after 49 and 56 days of yogurt storage in the S1 sample, as were the values for the hardness and adhesiveness of the same samples of yogurt (Table 1). This may suggest that the gel network of these yogurts stabilized at this time, although the pH value continued to decrease (Table 2). It is known that the gel network structure is a relatively dynamic system composed of casein, denatured whey proteins, and calcium phosphate crosslinks that are affected by many chemical and technological factors (i.e. dry matter content, enzyme activity, heat treatment, incubation temperature, pH) (Lee and Lucey, 2010). These observed changes are attributed to the enzyme which crosslinks milk protein. The addition of the enzyme to the milk and the time point of this addition significantly affected syneresis and, therefore, the WHC value. Samples with the smallest syneresis and the highest WHC were not characterized by the highest pH value.

The milk was inoculated with the same quantity of yogurt starter culture; thus, the population of lactobacilli averaged 8.0 ± 0.3 log CFU/mL before the fermentation, and was in the range of 8.0–8.2 log CFU/mL in fresh yogurt (Table 3); however, this did not depend significantly on the sample type (*p *>* 0.05*), demonstrating that the presence of mTGase had no effect on the biological activity of bacterial cells during fermentation. Moreover, the difference in the number of lactobacilli was not significant, either before or after fermentation (*p *>* 0.05*). A gradual, but significant, reduction in lactobacilli populations was observed throughout the refrigerated storage of yogurt samples (*p *<* 0.05*). Statistical analysis showed that there were no significant differences in the number of viable lactobacilli cells between the test samples (*p *>* 0.05*). This demonstrates that the presence of mTGase had no significant effect on the viability of bacterial cells in any yogurt. The *S. thermophilus* population did not change significantly as a result of fermentation (*p *>* 0.05*). However, the streptococcal populations differed significantly in the control yogurt and S2 samples (Table 3). Moreover, an impact of the presence of mTGase on the population of streptococci was noticed during the 56 days of storage, when a reduction in bacterial cell population was observed. This effect was not clear although it was significant (*p *<* 0.05*). Changes in the viability of streptococci were similar through days 21–28 of storage. However, on day 56 of refrigerated storage, S3 samples contained significantly more streptococcal cells than the control samples. In comparison, the S1, S2 and S3 samples did not differ significantly from each other in terms of streptococcal populations.

**Table 3:** Lactic acid bacteria count in yogurt samples

Storage time (day)	*Lb. delbrueckii* subsp. *bulgaricus* count (log CFU/ml)							
	C samples		S1 samples		S2 samples		S3 samples	
	Mean	± SD	Mean	± SD	Mean	± SD	Mean	± SD
0	8.0	± 0.22^a A,B^	8.1	± 0.16^a A,B,C^	8.2	± 0.42^a A^	8.2	± 0.33^a A^
7	8.2	± 0.14^a B,C^	8.0	± 0.60^a A,B,C^	8.0	± 0.83^a A^	8.1	± 0.78^a A^
14	8.3	± 0.17^a C^	8.5	± 0.30^a C^	8.1	± 0.32^a A^	8.3	± 0.41^a A^
21	8.3	± 0.38^a C^	8.4	± 0.27^a C^	8.2	± 0.27^a A^	8.4	± 0.26^a A^
28	8.2	± 0.37^a B,C^	8.4	± 0.29^a B,C^	8.3	± 0.27^a A^	8.3	± 0.26^a A^
35	8.1	± 0.42^a A,B,C^	8.2	± 0.30^a A,B,C^	8.2	± 0.27^a A^	8.1	± 0.28^a A^
42	8.1	± 0.34^a A,B,C^	8.0	± 0.21^a A,B^	8.0	± 0.30^a A^	8.2	± 0.38^a A^
49	7.7	± 0.52^a A^	7.9	± 0.21^a A^	7.8	± 0.35^a A^	8.2	± 0.32^a A^
56	7.8	± 0.56^a A,B^	7.9	± 0.21^a A,B^	7.7	± 0.35^a A^	8.0	± 0.39^a A^

Storage time (day)	*Str*. *thermophilus* count (log CFU/ml)							
	C samples		S1 samples		S2 samples		S3 samples	
	Mean	± SD	Mean	± SD	Mean	± SD	Mean	± SD
0	8.9	± 0.04^a A^	8.8	± 0.14^a,b A^	8.6	± 0.27^b A,B^	8.8	± 0.23^a,b A^
7	8.7	± 0.32^a A,B,C^	8.7	± 0.30^a A,B^	8.5	± 0.28^a A,B,C^	8.8	± 0.12^a A^
14	8.8	± 0.23^a A,B^	8.7	± 0.20^a A^	8.4	v0.30^b A,B,C^	8.7	± 0.26^a,b A^
21	8.7	± 0.31^a A,B,C^	8.5	± 0.20^a,b A,B^	8.4	± 0.41^b A,B,C^	8.7	± 0.27^a,b A^
28	8.7	± 0.10^a A,B,C^	8.6	± 0.28^a A,B^	8.4	± 0.42^a A,B,C^	8.6	± 0.27^a A,B^
35	8.4	± 0.44^a C^	8.5	± 0.25^a A,B^	8.7	± 0.18^a B^	8.5	± 0.21^a A,B,C^
42	8.5	± 0.32^a B,C^	8.3	± 0.25^a B,C^	8.4	± 0.30^a A,B,C^	8.2	± 0.22^a B,C^
49	7.9	± 0.11^a D^	8.1	± 0.30^a C^	8.1	± 0.34^a A,C^	8.2	± 0.22^a C^
56	7.7	± 0.32^a D^	8.1	± 0.30^a,b C^	7.9	± 0.47^a,b C^	8.2	± 0.22^b C^

C samples—obtained from milk with skimmed milk powder added at 12 h prior to fermentation and stored at 6 °C until fermentation, then a starter culture was added to milk prior to fermentation

S1 samples—obtained from milk with skimmed milk powder and the mTGase added at 12 h prior to fermentation and stored at 6 °C until fermentation, then a starter culture was added to milk prior to fermentation

S2 samples—obtained from milk with skimmed milk powder added at 12 h prior to fermentation and stored at 6 °C until fermentation, then mTGase and a starter culture were added immediately prior to fermentation

S3 samples—obtained from milk with skimmed milk powder and mTGase added at 12 h prior to fermentation and stored at 6 °C until fermentation, then milk was heated to 80 °C and a starter culture was added after cooling down to 45 °C before the fermentation process

^a, b, c^Significant differences in each row among the means (for *p* < 0.05)

^A, B, C, D^Significant differences among the means (in each column for each parameter) (for *p* < 0.05)

Mean ± SD = mean values ± standard deviations of six experiments

**Fig. 1:**
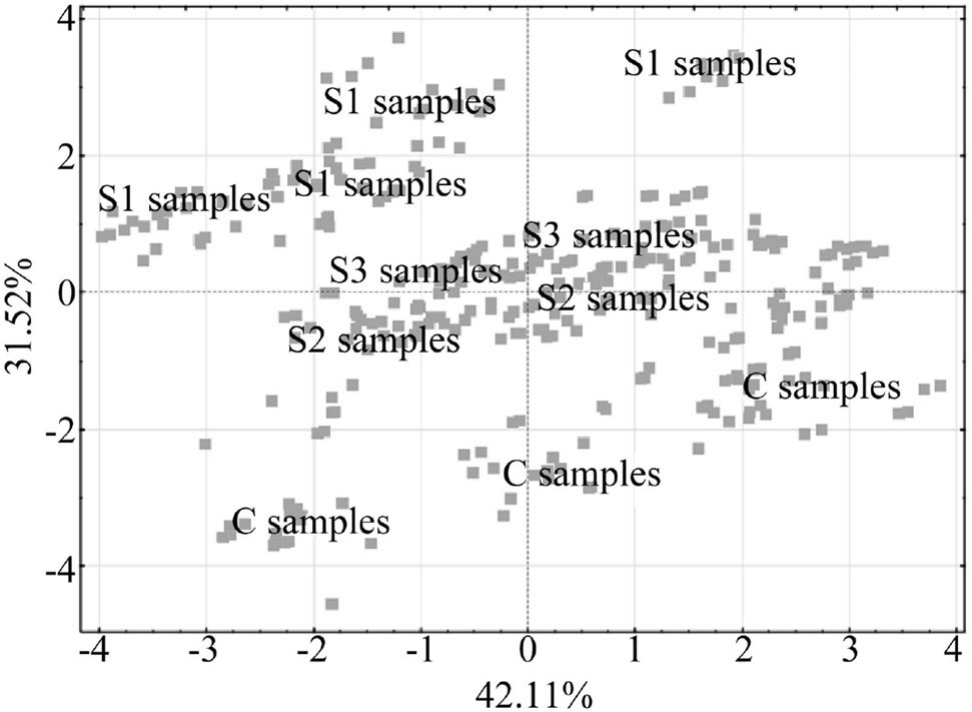
The principal components analysis (PCA) of cases on the basis of the first (42.11%) and second (31.52%) major components. C samples—obtained from milk with skimmed milk powder added at 12 h prior to fermentation and stored at 6 °C until fermentation, then a starter culture was added to milk prior to fermentation. S1 samples—obtained from milk with skimmed milk powder and the mTGase added at 12 h prior to fermentation and stored at 6 °C until fermentation, then a starter culture was added to milk prior to fermentation. S2 samples—obtained from milk with skimmed milk powder added at 12 h prior to fermentation and stored at 6 °C until fermentation, then mTGase and a starter culture were added immediately prior to fermentation. S3 samples—obtained from milk with skimmed milk powder and mTGase added at 12 h prior to fermentation and stored at 6 °C until fermentation, then milk was heated to 80 °C and a starter culture was added after cooling down to 45 °C before the fermentation process

**Fig. 2:**
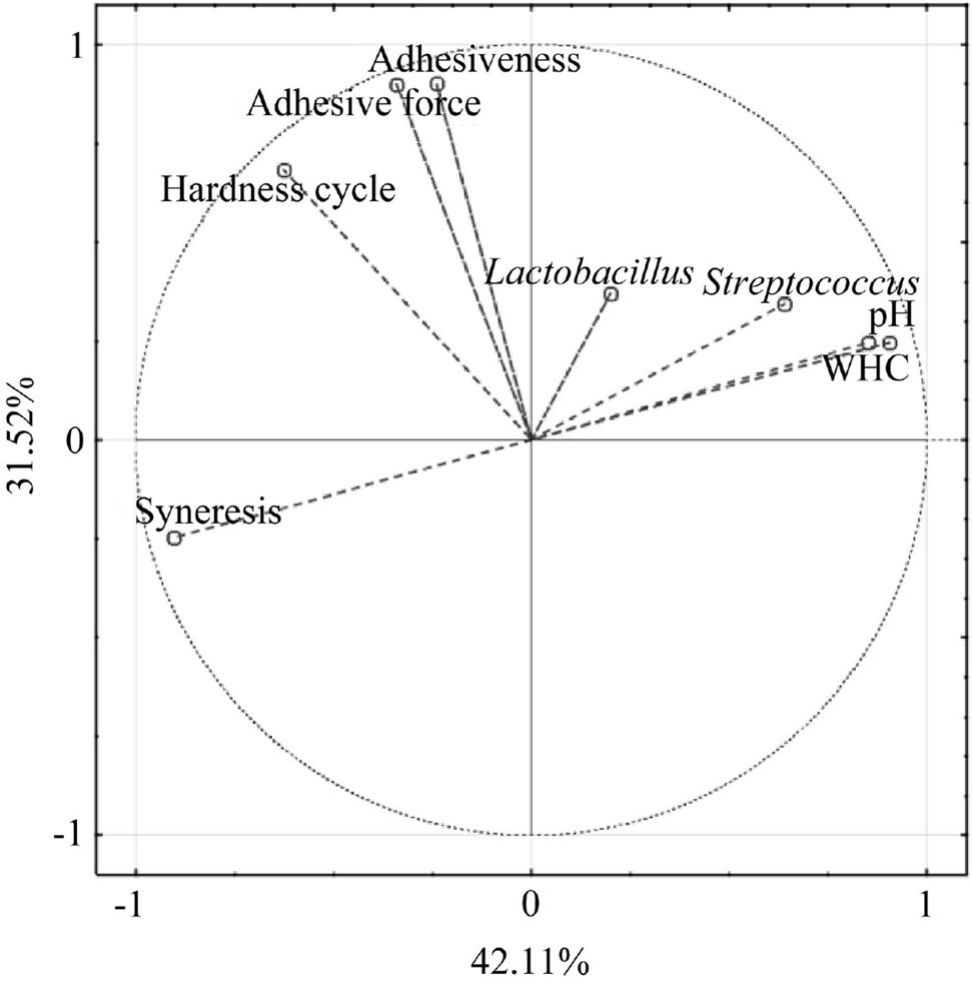
Projection of variables on the first plan given by principal component analysis (PCA). The first major component (42.11%) is first axis given by PCA 1; and second major component (31.52%) is second axis given by PCA 2

PCA was undertaken to illustrate the differences between all of the studied samples. A model with two main components that explain 73.62% of the total variation (Fig. 1) was chosen, and allowed the extraction of examples belonging to S1 samples. The strong influence of variables such as cycle hardness, adhesive force, and adhesiveness on the selected main components (Fig. 2) was evident in the separation of S1 examples from other samples. The PCA allowed the simultaneous grouping of the studied cases into three distinctive groups: S1 samples, a mixed group of S2 and S3 yogurts, and control samples (C samples in Fig. 1). This grouping reflects the impact of enzymes on the structural characteristics of the final product. Based on the distribution of examples, the most important structural changes were observed in S1 samples, whereas the characteristics and properties of S2 and S3 yogurts were similar to those of the control samples.

The mTGase activity leads to an increased density of the network in the yogurt gel and, thus, reduces the pore size of this network due to the polymerization of caseins and whey proteins (Lee and Lucey 2010). Fortification of total milk solid content significantly influences the properties of yogurt (Pakseresht et al., 2017). Therefore, enzymatic protein crosslinking could be applied in yogurt production instead of stabilizers. Casein is a more suitable substrate for mTGase because of its open structure, and its globular conformation of whey proteins prevents the formation of covalent links between caseins by enzymatic reactions (Bonisch et al., 2007; Sanli et al., 2011). These structural changes result in a reduction of syneresis, which may have an effect on the viability of lactic acid bacteria.

Our experiments have shown that the presence of mTGase significantly affects syneresis and the WHC of yogurt gel; however, there was no association with the viability of lactobacilli or streptococci. Syneresis is not desirable in yogurts. The literature contains plenty of evidence that TGase has a positive impact on reducing syneresis and improving the WHC of yogurts (Kuraishi et al., 2001; Lorenzen 2007; Vargas et al., 2008). Lorenzen et al. (2002) found that pore size is decreased and syneresis is reduced when milk is treated with TGase. Our experiments have proved that both the presence and duration of the activity of mTGase affect syneresis and WHC, and that this impact becomes stronger with storage time. Although Ozer et al. (2007) found that changes in the amount of whey separated from yogurt stored for 21 days did not depend on enzymatic treatment, this was observed in our experiments when yogurts were stored for 56 days. We found no correlation between the changes in the lactic acid bacteria population or pH and the changes in WHC or syneresis in yogurts. Thus, it can be concluded that the water bound in a yogurt gel cross-linked with mTGase was neither less nor more available for the bacterial cells; furthermore, it did not affect the concentration of hydrogen ions in the yogurt. This is confirmed in the observations of Moon and Hong (2003): that a more compact gel microstructure with smaller pores in the structure of yogurt curd results in enhanced trapping of water in this network. Sanlı et al. (2011) observed similar syneresis values in yogurts with inactive TGase to those in samples without TGase, and this did not change over the storage period. Similar results to those of this study were obtained by Ozer et al. (2007), but only in the first week of storage. Then, starting from day 28 of the experiments, a clear effect on syneresis and WHC was observed to result from the addition and duration of activity of the enzyme in milk.

Our analysis of selected textural properties showed the impact of the presence and duration of mTGase activity on yogurt stored in a refrigerator for 56 days. Textural properties are a useful source of information about the characteristics of crosslinking protein gels. The network of yogurt gel becomes increasingly compacted and regular as a result of TGase action (Lauber et al., 2000a; 2000b). This leads to improved gelling properties and hardness (Gauche et al., 2009). Yuksel and Erdem (2009) observed this effect not only in fresh yogurt, but also during refrigerated storage. They explained that TGase does not lose its activity during storage and acted even at low temperatures. This explanation may be true if TGase activity is not lost due to decreases in pH value, as was argued by Bonisch et al. (2007). We observed differences in the properties of the texture of yogurts prepared from milk treated with mTGase in various combinations. The direction of these changes may suggest that not only is enzymatic activity important, but so are other changes that take place in the yogurt–even the gradual decrease in populations of lactic acid bacteria and the lysis of their cells, the decrease in pH value, and the increase in the syneresis value (Zaręba et al., 2008a; 2008b; 2014). Moreover, mTGase becomes inactive in yogurt stored in the refrigerator for a long time, as concluded Bonisch et al. (2007); however, further changes occur, and these are induced by the presence of the enzyme in the milk both before and during fermentation. This conclusion allows a comparison of the results obtained for S1 yogurts, wherein the enzyme has been active in milk flora for a long time, and those from other samples, where the enzyme was inactive or was active for only a short time prior to, or during, fermentation. Furthermore, a comparison of the observations of Gauche et al. (2009) and the results produced by Bonisch et al. (2007) could provide indirect evidence of this hypothesis. The first of the cited research groups demonstrated a significant increase in almost all textural parameters of yogurt that was produced from milk to which TGase had been added before the fermentation. The second team of researchers used TGase during fermentation and did not find any effect of this enzyme on yogurt texture. Similar to our experiments, Domagała et al. (2013) showed that yogurt with TGase had higher values for textural parameters compared to the control yogurt.

The main effect of TGase in the production of yogurt was to strengthen the curd protein matrix and its mechanical resistance and improve texture with or without enrichment in non-fat dry matter. However, the literature reports a negative impact of enzymes on the population of lactic acid bacteria. Most microbiologists would agree that a < 1 log reduction is not biologically significant, despite the fact that there may be a statistical difference. The addition and activity of mTGase did not affect lactobacilli populations, but had a variable effect on streptococcal populations in yogurts. Intermolecular polymerization of milk proteins catalyzed by mTGase leads to significant changes in the microstructure of yogurt gel (Lee and Lucey, 2010). However, it is known that the reactions of free amino acids and peptide additions to proteins via covalent bonds catalyzed by TGase can lead to decreases in the growth of yogurt bacteria, as was observed by Ozer et al. (2007). Our experiments, however, did not confirm the results obtained by Ozer et al. (2007), demonstrating that the effect of mTGase concentration increases and the number of *L. delbrueckii* subsp. *bulgaricus* decreases during the storage of yogurts. Our results are more in agreement with the observations made by Farnsworth et al. (2006), who found no significant difference in the population of probiotic cultures of *Lactobacillus* and *Bifidobacterium* in yogurts, compared to control samples. Similar observations were made by Mituniewicz-Małek et al. (2014). In addition, there are reports that the use of mTGase does not have a negative impact on yogurt fermentation time (De Jong et al., 2003). As our observations proved, mTGase may affect the streptococcal population, although this impact is not unambiguous with respect to the activity of the enzyme both before and during the fermentation. The observed differences in the level of streptococci were related to differences in the pH values of yogurts. Moreover, we may assume that the variability of the streptococcal population in the tested yogurt samples may depend on the fact that they are aerobic bacteria, unlike anaerobic lactobacilli. However, further studies are required to confirm this assumption.

A change in the acidity of fermented milk is normally associated with the level of viable bacterial cells of starter lactic acid bacteria and their viability during storage (Domagała et al., 2007; Sanli et al., 2011). Ozer et al. (2007) showed that the increase in the acidity of yogurt was reduced with increasing doses of mTGase, and they attributed this to growth inhibition of lactic acid bacteria. They found the highest pH in fresh yogurt samples with mTGase, and noted that the pH value similarly decreased during storage of all samples of yogurt.

The presence of mTGase and the duration of its activity in milk prior to fermentation, both, affect syneresis and the WHC of yogurts; however, our experiments demonstrated that the quality characteristics of yogurts were dependent on the step in the production process at which mTGase was added to milk. A clear effect of the enzyme is observed in terms of the textural features of yogurts. The hardness, adhesive force, and adhesiveness of yogurts are determined by the duration of enzymatic activity in milk before fermentation. The pH value of yogurts gradually decreases in a significant manner during refrigerated storage, regardless of the presence of mTGase. The addition of mTGase had no effect on *L. delbrueckii* subsp. *bulgaricus* count, but had a variable effect on the population of *S. thermophilus* in yogurts during fermentation or storage at 6 °C for 56 days, depending on the duration of enzymatic activity. The observed differences in the level of streptococci were related to differences in the pH values of yogurts. An improvement was observed in the quality of all set-style yogurt samples with mTGase addition, but especially in those samples obtained from milk with SM powder, and where the mTGase was added to milk just before the fermentation. The selected parameters of yogurt thus obtained were better than those of yogurt obtained from milk with SM powder and mTGase added 12 h before the fermentation and heated to 80 °C just before the fermentation.
